# Potential effect of bio-surfactants on sea spray generation in tropical cyclone conditions

**DOI:** 10.1038/s41598-020-76226-8

**Published:** 2020-11-04

**Authors:** Breanna Vanderplow, Alexander V. Soloviev, Cayla W. Dean, Brian K. Haus, Roger Lukas, Muhammad Sami, Isaac Ginis

**Affiliations:** 1 Halmos College of Arts and Sciences, Nova Southeastern University, Dania Beach, FL USA; 2 University of Miami, Miami, FL USA; 3 University of Hawaii, Honolulu, HI USA; 4 Ansys, Inc., Houston, TX USA; 5 University of Rhode Island, South Kingstown, RI USA

**Keywords:** Physical oceanography, Fluid dynamics

## Abstract

Despite significant improvement in computational and observational capabilities, predicting intensity and intensification of major tropical cyclones remains a challenge. In 2017 Hurricane Maria intensified to a Category 5 storm within 24 h, devastating Puerto Rico. In 2019 Hurricane Dorian, predicted to remain tropical storm, unexpectedly intensified into a Category 5 storm and destroyed the Bahamas. The official forecast and computer models were unable to predict rapid intensification of these storms. One possible reason for this is that key physics, including microscale processes at the air-sea interface, are poorly understood and parameterized in existing forecast models. Here we show that surfactants significantly affect the generation of sea spray, which provides some of the fuel for tropical cyclones and their intensification, but also provides some of the drag that limits intensity and intensification. Using a numerical model verified with a laboratory experiment, which predicts spray radii distribution starting from a 100 μm radius, we show that surfactants increase spray generation by 20–34%. We anticipate that bio-surfactants affect heat, energy, and momentum exchange through altered size distribution and concentration of sea spray, with consequences for tropical cyclone intensification or decline, particularly in areas of algal blooms and near coral reefs, as well as in areas affected by oil spills and dispersants.

## Introduction

Predicting intensities of tropical cyclones, especially their rapid intensification, remains a daunting challenge despite advances in model forecasting through increased computer power and improved observational data systems. While cyclone track and intensity prediction of longer time scales has substantially improved, there has been less improvement in the accuracy of forecasting the rapid intensification of tropical cyclones within a 24-h period since the 1990′s^1–3^. Intensity error from the best available model has decreased by only 1–2% per year between 1989 and 2012^2^. Tropical cyclones Charley 2004, Wilma 2005, Humberto 2007, Maria 2017, and Dorian 2019 all intensified prior to landfall, devastating unprepared communities due to rapid intensification that was missed by tropical cyclone forecast models. Effective tropical cyclone forecasting is highly complex and must account for many processes in both the atmosphere and ocean^4^. Recent model improvements include focusing on the inner-core structural changes of the storm^5^, implementing cloud-resolving models^6^, and increasing model resolution to below 1 km^7^. In addition to improvements to the atmospheric component, concepts of ocean heat content^8,9^, barrier layer^10^, and air-sea interface^11^ have recently been introduced into the consideration of tropical cyclone physics.

Intensity forecasting remains a challenge in part due to a lack of understanding of key physics that contribute to intensity and intensification of tropical cyclones. This includes cloud microphysics. It has been predicted that aerosols serving as cloud condensation nuclei intensify the tropical cyclone if the they penetrate the central clouds of the storm but weaken it if they penetrate the clouds at the storm periphery^12^.

Microscale processes at the air-sea interface still need to be adequately parameterized in existing models. An effort to include bulk parameterization of the air-sea momentum flux, which was found to be of key importance to sea surface wind speeds, enhanced the parameterization of the wind-pressure relationship of major tropical cyclones in prediction models^13^.

Lee et al. ^14^ considered the wind-dependent drag coefficient based on the air-sea microphysics^11^, which led to an improvement in prediction of rapid intensification within 24 h by 16%. Overlooking key physics in models often leads to inaccurate hurricane intensity predictions, further leading to erroneous warnings and evacuations that may cost lives. Therefore, it is essential to improve representation of air-sea fluxes and their effect on tropical cyclone intensity into future prediction models in order to increase forecasting accuracy. Improved tropical cyclone prediction is particularly critical during pandemics, such as the COVID-19 outbreak, where poor prediction could cost lives if unneeded sheltering of large groups occurs. Here we focus on sea spray, which is another potentially important factor in tropical cyclone dynamics.

Under tropical cyclone conditions, spray droplets contribute to the heat and momentum transfer between the ocean and atmosphere^15–18^. Kepert et al.^19^ and Peng and Richter^20^ noted the following fundamental issues that relate to spray effects: the sea spray generation function, the feedback by which spray droplets modify the environmental conditions, and parameterization of the thermodynamic effects of sea spray for tropical cyclone models. These problems remain due to the complexity of the air-sea interaction process during tropical cyclones and difficulties in direct observations during extreme conditions. Only a few experimental or observational studies attempted to measure the heat fluxes in tropical cyclone conditions (*e.g.,* Drennan et al.^21^, Zhang et al.^22^). Tropical cyclone analysis is generally based on indirect measurements^24,25^ or laboratory experiments^25,26^.

During tropical cyclones, the atmosphere and ocean are strongly coupled. The air-sea interface controls momentum, heat, mass, and energy exchange between the ocean and atmosphere. Tropical cyclones gain heat energy, as well as transfer momentum and kinetic energy to the ocean, through the air-sea interface. In high wind conditions under tropical cyclones, a two-phase environment is created. In this environment, spray droplets are generated by white caps on breaking waves and in the process that resembles the Kelvin–Helmholtz (KH) instability^11,27,28^. In tropical cyclones wave-breaking whitecaps, which are a mixture of bubbles and spray, only cover ~ 4%, while the ‘white out’ associated with foam and spray streaks covers ~ 96% of the sea surface^29^. Whitecapping with spray and bubble formation occurs once wind speeds exceed 7–9 m s^−1^, but the formation of a continuous two-phase environment is only observed when wind speeds are above ~ 30 m s^−1^.

Under light winds, the KH instability of the air–water interface contributes to surface wave generation in the gravity‐capillary range^30^. Under wind speeds above 4–5 m s^−1^ short wavelets steepen and break internally, causing ‘microscale wave breaking’, which does not disrupt the air-sea interface enough to eject spray^31–34^. Under tropical cyclone force winds, the gravity and surface tension forces are overcome by pressure fluctuations due to KH instability in the air flow, which disrupt the air-sea interface leading to sheets, fingers, and intense sea spray generation^28,35^.

In laboratory experiments, radii of sea spray have been observed in the range of less than 1 μm to up to 6 mm^36,37^. Small sea spray particles, which are typically sub-micrometer to tens of micrometers in diameter, are mostly generated by bursting air-bubbles^38,39^, which produce film and jet spray droplets. Film droplets typically range from 0.5 to 5 μm and jet droplets from 3 to 50 μm. Another mechanism of spray generation is ‘bag-breakup’ fragmentation^40^, in which bag-like pieces of water inflate and then quickly burst into spray droplets.

Larger spray particles, above 20 μm (spume), are produced by “tearing of water” from wave crests^41^. Koga^27^ and Veron et al.^37^ found that near the wave crest, where the wind stress is usually the highest, small convoluted projections of the water surface develop and break up to form spume particles. The projections resemble the KH type instability at an interface with a very large density difference, which is characterized by strong asymmetry^35^. The majority of the action occurs on the air side of the air-sea interface; in fact, the KH instability generates spray and spume in the air but very few bubbles are produced in the water. At the same time, air bubbles are mostly associated with whitecaps produced by longer breaking waves interacting with shorter, steeper gravity waves driven by local wind fluctuations^42^.

The spray particles produced by these mechanisms are either entrained in the turbulent air flow and evaporate, or they return to the sea surface^36,43^. Small spray droplets are typically entrained in the turbulent air flow, eventually evaporate, and thus may not contribute much to the enthalpy flux into a tropical cyclone due to spray negative feedback phenomenon^20^. Larger spray droplets (spume) are mostly ‘re-entrant’ spray, which significantly contribute to the enthalpy flux to tropical cyclones^36^.

One of the factors affecting the stability of the air-sea interface are surface-active materials (surfactants). Surfactants are often produced by marine organisms such as phytoplankton, zooplankton, zooxanthellae, and bacteria. Surfactants may also appear on the sea surface during oil spills and through the use of dispersants. Surfactants alter surface tension, dampen short gravity-capillary waves, produce slicks, and reduce the air-sea drag coefficient under low wind speed conditions. To our best knowledge the effect of surfactants on spray size distribution and tropical cyclone intensity has not yet been studied.

The coverage, concentration, and composition of slicks vary depending on wind, sea conditions, and time. When wind speeds are above 7 to 10 m s^−1^, breaking waves disrupt slick formation and overwhelm the effects of surfactants^44^. The effects of surfactants once again become important under tropical cyclone conditions due to sea spray. Notably, the size distribution of sea spray is expected to depend on the presence of surfactants. During high wind speed conditions surfactants are brought to the surface by turbulence and air-bubbles with increased mixing in the water column, enhanced by upwelling under the tropical cyclone^45–47^.

Consequently, we show here that surfactants might be a factor in tropical cyclone intensity, including rapid intensification and decline, through the altered size distributions of sea spray. Our experiments at a state-of-the-art laboratory facility and using a new computational fluid dynamics (CFD) method have resolved spray size distributions starting from a 100 μm radius and help to address this problem. The CFD model has been verified with laboratory experiments in the range of spume size distribution. Using this model, we examine the spume concentration for Category 1 (75–95 mph), Category 3 (111–129 mph), and Category 5 (157–195 mph) tropical cyclone conditions to show substantial increase of spume concentration in the presence of surfactants. We anticipate that bio-surfactants extracted from deeper in the ocean affect heat, energy, and momentum exchange through altered size distribution and abundance of sea spray, with possible consequences for tropical cyclone intensification or decline.

The challenge of predicting tropical cyclone intensity and intensification includes multiple factors. Tropical cyclones require certain environmental conditions to develop and intensify. These include warm sea surface temperature, high ocean heat content, high relative humidity, low vertical wind shear, and an initial vortex at some distance from the equator^48–51^. Presence of the salt-stratified barrier layer^10^ is relatively new, but important factor in the problem of tropical cyclone prediction (see, *e.g*., Grodsky et al.^52^, Kao and Lagerloef^53^). Bio-surfactants have not been previously considered as a factor in tropical cyclone thermodynamics. In this paper we report our computational and laboratory results of the effect of surfactants on the size distribution of spray that might alter air-sea fluxes of heat and momentum and potentially affect tropical cyclone intensity.

## Results and discussion

Laboratory experiments involving tropical cyclone force wind speed conditions were conducted at the University of Miami SUrge STructure Atmosphere INteraction Facility (SUSTAIN). Surfactants, either oleic acid or oleyl alcohol, were diluted in 95% ethanol. Experiments were conducted with and without surfactants. In the experiment including release of surfactants, 60 ml of the surfactant solution was added to the upwind side of the tank using a syringe.

Conspicuously, Brockmann et al.^54^ experimented with artificial release of surfactants (oleyl alcohol) on the ocean surface from a helicopter. They found that the concentration of this surfactant at the surface was 0.02 mol/l. For comparison, our laboratory experiment used an order of magnitude smaller concentration of surfactant, which still had a prominent effect on the spray generation.

**Figure 1:**
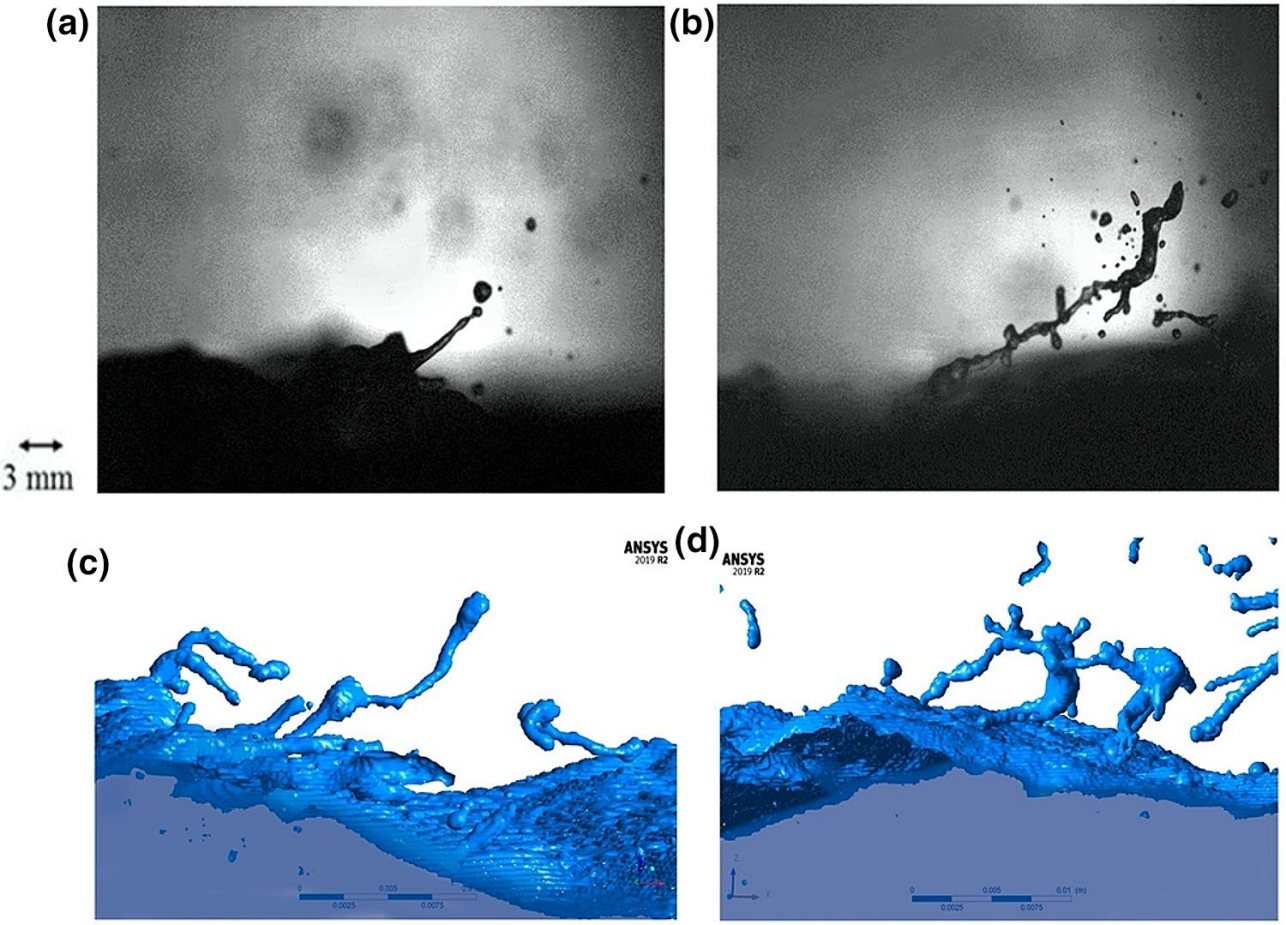
Disruptions of the air–water interface under Category 1 (U_10_ = 40 m s^−1^) tropical cyclone conditions from the laboratory and computational data. (**a**) Finger-like structure formed in the absence of surfactants during the laboratory experiment. (**b**) Branch-like structure formed in the presence of surfactants during the laboratory experiment. (**c**) Finger-like structure formed in the absence of surfactants in the VOF-to-DPM. (**d**) Branch-like structure formed in the presence of surfactants in the VOF-to-DPM.

The laboratory experiments revealed visible differences of spray generation when surfactants were either absent or present (Fig. 1a,b). The Volume of Fluid to Discrete Phase Model (VOF-to-DPM) transition model in ANSYS Fluent CFD code reproduced similar patterns shown as an isosurface (cells of a specific constant value) of the water surface in Fig. 1c,d. In the absence of surfactants, the mechanism for spume generation resembles a finger-like structure, caused by the KH instability at an interface with a large density difference^11, 27,35^ (Fig. 1a,c). When surfactants are present, altering the sea surface through reduced surface tension, the generation of spume occurs differently. Figure 1b,d show branch-like formation of spray. This ultimately leads to a different size distribution with a higher concentration of droplets.

**Figure 2:**
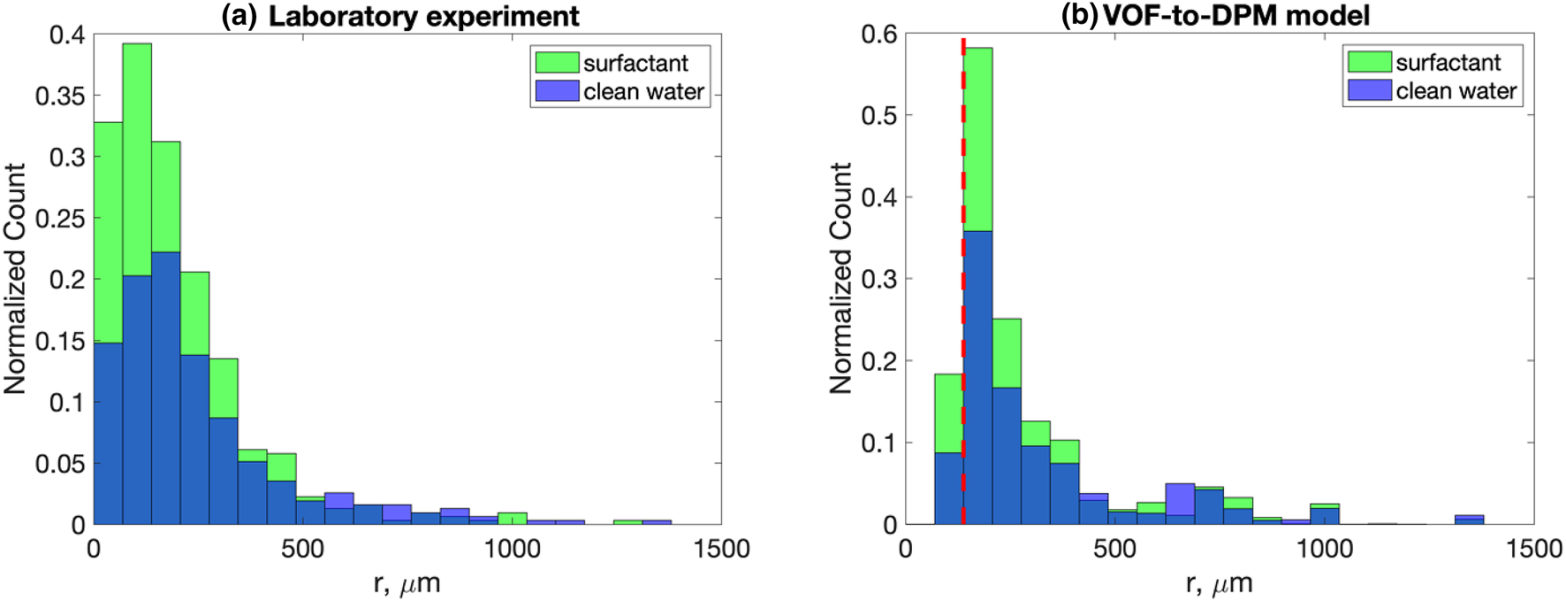
Histograms showing the spray radius distribution under Category 1 tropical cyclone conditions for (**a**) the laboratory experiment and (**b**) the VOF-to-DPM. The vertical dashed line shows the lower resolution limit of the model. The y-axis scale for the model is substantially larger than for the laboratory experiment (see explanation in the text). For this reason, in each subplot, the vertical axis has been normalized by the total number of samples for the clean water case. The total number of spray particles before normalization in bins 1–21 for each panel are as follows: laboratory experiment clean water 311, surfactant 491; VOF-to-DPM model clean water 9534, surfactant 13,826.

We compared the Category 1 tropical cyclone wind-forced VOF-to-DPM results with a 4 Nm^−2^ wind stress at the top of the numerical tank to the laboratory experiment with comparable wind stress (extrapolated U_10_ = 40 m s^−1^ wind speed used at SUSTAIN). The model shows a much higher amount of spray in both surfactant and clean water cases than the laboratory experiments. This is because in the laboratory experiment, the images used for data analysis were taken at one plane of the tank, so a large amount of spray was unaccounted for in these results in comparison to a 3D model that accounts for spray throughout the domain. For comparison, we have normalized laboratory and numerical experiments by the total count of spray particles. In each subplot of Fig. 2, the vertical axis has been normalized by the total number of samples for the clean water case. The total number of spray particles for each panel is given in the figure caption.

The laboratory experiment was conducted using saltwater (~ 33 psu) while the model fluid was set to freshwater. The Nayar et al.^55^ parameterization shows the difference in surface tension between sea and fresh water on level of 1.4%. The density difference is on the level of 2.5%, and molecular viscosity about 7.5%. These cannot explain the significant difference in foaming between salt and freshwater observed in nature. Katsir and Marmur^56^ explained that saltwater is foamier than freshwater because of the ionic effect related to different coalescence properties of air-bubbles in sea and fresh water. The KH instability produces mostly large spray particles (spume), which is not directly related to bubble dynamics.

The initial mesh resolution in the numerical domain is 2000 μm. Dynamic mesh adaption locally increases mesh resolution allowing the VOF-to-DPM to accurately resolve spray radius distributions starting from ~ 100 μm (Fig. 2a). Laser imaging techniques used in the laboratory experiment resolved a spray radius starting from 30 μm (Fig. 2b) and were used to estimate spatial resolution of the model.

For Category 1 tropical cyclone conditions, the laboratory experiment revealed a 39% increase in spray concentration in the range of radii from 100 to 500 μm when surfactants were present (Fig. 2a). The model indicated a 34% increase in spray between clean water and surfactant presence for the same conditions and the same range of radii (Fig. 2b). Spray radius distributions above 500 μm appear to be more intermittent and are less informative in the comparison between laboratory and model results.

**Figure 3:**
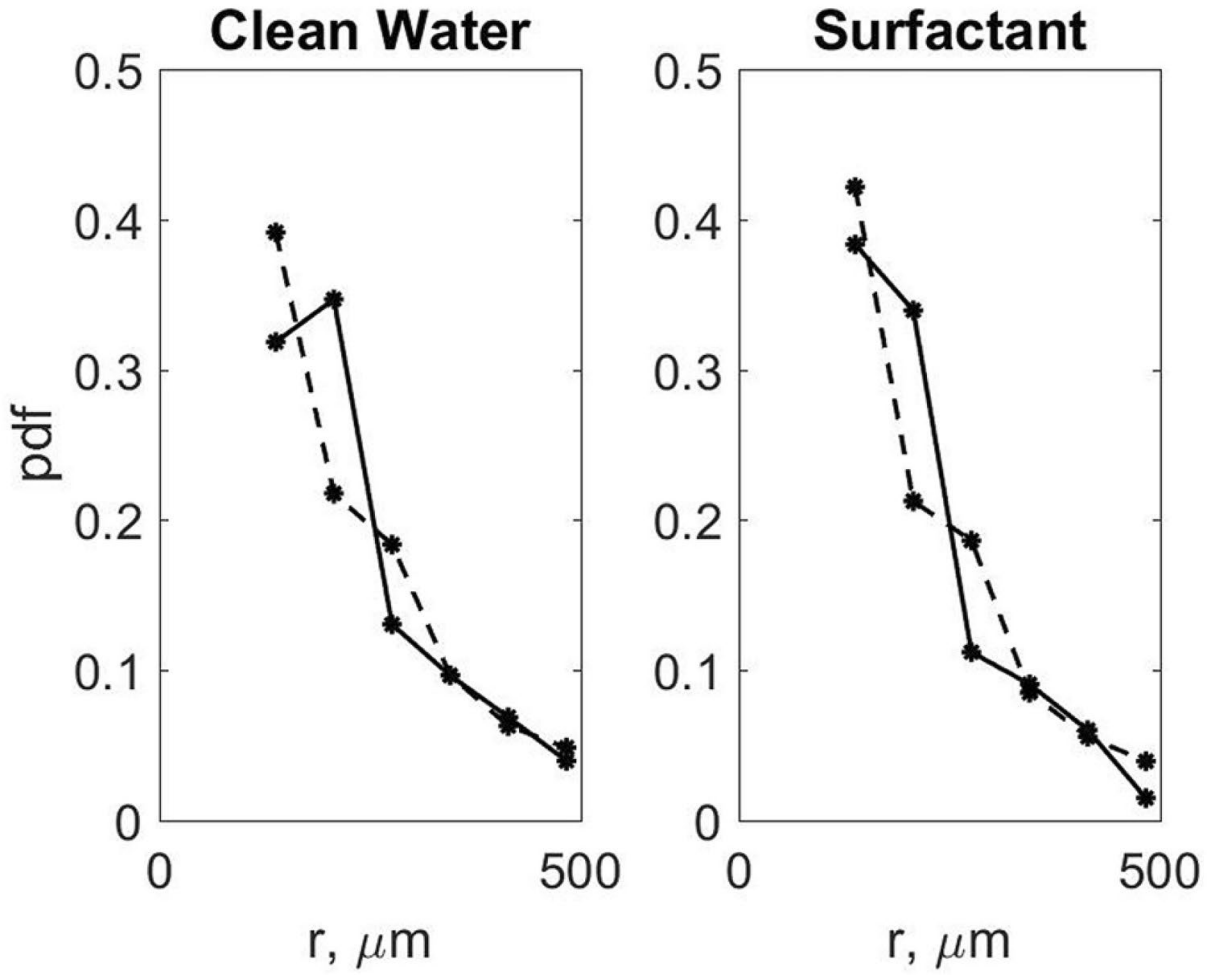
Probability density of spray radius distributions for clean water and surfactants in the laboratory experiment (dashed line) and VOF-to-DPM (continuous line) under Category 1 tropical cyclone conditions.

Figure 3 shows validation of the model with the laboratory data using the probability density function (pdf) of spray radii. For both clean water and surfactant cases there is good consistency between the model results and laboratory experiment data.

**Figure 4:**
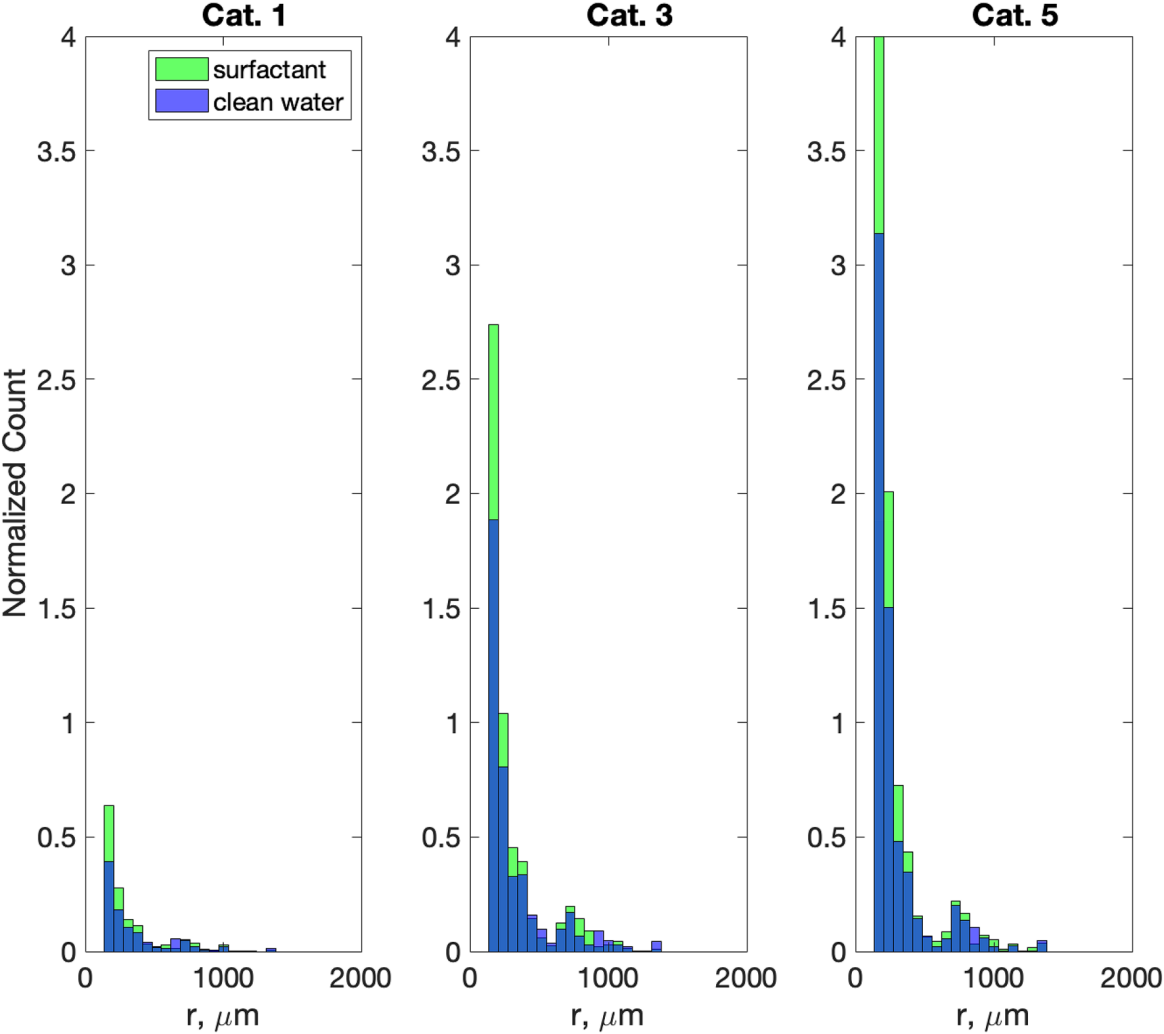
Spray radius distributions for Category 1 (4 Nm^−2^), 3 (10 Nm^−2^), and 5 (20 Nm^−2^) tropical cyclone conditions in the VOF-to-DPM. Scales below 100 μm are not completely resolved (see Fig. 2) and have been removed from graphs. To demonstrate the increase of spray generation with wind due to the presence of surfactants for all tropical cyclone categories, the vertical axis has been normalized by the total number of samples for the Category 1 clean water case. The total number of spray particles before normalization in bins 3–21 for each panel are as follows: Cat. 1 clean water 8701, surfactant 12,073; Cat. 2 clean water 36,942, surfactant 48,074; Cat. 5 clean water 55,193, surfactant 74,104.

Using the validated VOF-to-DPM, we calculated spray radii distributions for Category 1, 3, and 5 tropical cyclones (Fig. 4), which would be difficult to do in the laboratory experiment. Overall, spray generation increased with wind speed, and in all tropical cyclone categories, surfactants caused a 20–34% increase of spray concentration within the spray radii range from 100 to 500 μm.

Sea spray provides additional fuel for tropical cyclones and their intensification. Spume appears to be a significant factor in generating the enthalpy flux to tropical cyclones due to its short residence time and is less affected by the spray feedback effect^20,57^. Due to the spray feedback effect, sub-micrometer and micrometer scale sea spray particles, which have relatively large residence time, may not significantly contribute to the air-sea flux of enthalpy into tropical cyclones compared to spume.

The added momentum flux is due to the entire spectrum of spray sizes; however, the larger size droplets (spume) can produce a larger contribution to the added momentum flux because they have a larger mass (proportional to the radius cubed). Spray takes a part of the momentum flux from wind in the near-surface layer of the marine atmosphere, thus increasing drag^58^. This effect becomes prominent only in Category 3–5 tropical cyclones because spray stress is approximately proportional to wind speed to the fourth power^16^. The drag increase due to spume can lead to a rapid decline of the major tropical cyclones above 60 m s^−1^^11^. The reduction of the drag coefficient in the wind speed range from approximately 35–60 m s^−1^ observed in laboratory and field experiments can be explained by suppression of short gravity-capillary waves by the KH type instability of the air-sea interface^11,27,28^. Under certain environmental conditions, a combination of drag reduction from 35 to 60 m s^−1^ and drag increase above 60 m s^−1^ due to added spray stress contributes to the development of an aerodynamic drag well around a wind speed of 60 m s^−1^^43^.

In the presence of surfactants, the spume generation under tropical cyclone conditions is greater than in clean water (see Fig. 4). Therefore, as surfactants increase spray generation, they also increase the enthalpy flux to the atmosphere and consequently may affect tropical cyclone intensity. One problem in evaluating the effect of surfactants on tropical cyclones is that the abundance of bio-surfactants in the ocean is virtually unknown on a global scale. It is, however, well known that a small amount of surfactant can cover a large surface area^45^. Measurements of surfactants in the ocean are extremely rare. King et al.^59^ reported surface tension measurements in the coastal North Sea in the presence of surfactants in the range from 0.053 to 0.0681 N/m. In the model we set the surface tension reduction due to the effect of bio-surfactants to 0.054 N/m compared to the surface tension of clean water 0.072 N/m. This is within the range of the observations by King et al.^59^.

Sea spray influences the enthalpy coefficient (C_k_) and the drag coefficient (C_d_), which both depend on the spray in tropical cyclone conditions to some extent. A connection of spray to tropical cyclone intensity is consistent with the concept of maximum potential intensity^60–63^ (MPI), which estimates the upper limit of tropical cyclone intensity as follows:

1 $${V}^{2}=({k}^{*}-k)\frac{(\stackrel{-}{T}-{T}_{0})}{{T}_{0}}\frac{{C}_{k}}{{C}_{d}}$$

where V is wind speed, C_k_ the enthalpy coefficient, C_d_ the drag coefficient, k^*^ the saturation enthalpy at the sea surface, k the enthalpy, $\stackrel{-}{T}$ represents sea surface temperature^60^ (which later^62, 63^ was proposed to be treated as the pre-cyclone depth-averaged temperature), and T_0_ the outflow temperature at the top of the tropical cyclone. MPI is proportional to the ratio C_k_/C_d,_ thus controlling the maximum tropical cyclone intensity for given other variables. C_k_ may not strongly depend on wind speed for winds ${U}_{10}$ > 10 m s^−1^ at 10 m height^25^. The laboratory result from Jeong et al.^25^ was limited to the maximum equivalent neutral stratification wind speed of ${U}_{10}=40$ m s^−1^, which was in part corroborated by aircraft-based flux measurements^21^ and extended by Richter and Stern^24^ and Bell et al.^23^ to ${U}_{10}=70-75$ m s^−1^ using dropsonde data or by utilizing the conservation of azimuthally averaged absolute angular momentum, respectively. The laboratory experiment by Komori et al.^26^ conducted in the high-speed wind-wave tank at Kyoto University reported substantial increase in C_k_ above a 35 m s^−1^ wind speed, which could be due to intense spray generation.

The potential for rapid intensification is related to the difference between the current storm intensity and the MPI^14^. In the absence of external environmental factors that might inhibit intensification (ocean heat availability, large-scale shear, upper troposphere outflow temperature) the storm should intensify as fast as the moisture convergence and subsequent convective diabatic heating can feed back onto the surface circulation and subsequently the moisture convergence. There are both advective time scales (horizontally and vertically), as well as a gravity wave speed time scale. The feedback is positive, and as suggested above, the strength of the feedback is proportional to the thermodynamic imbalance. Since the MPI depends on C_d_ and C_k_, the thermodynamic imbalance is potentially dependent on the state of the air-sea interface and the presence of sea spray.

Several laboratory and field experiments reported the leveling off or decreasing of C_d_ above 30–35 m s^−1^ wind speeds^23,64–68^. Above 60 m s^−1^, sea spray (spume) could result in the increase of C_d_ due to entrainment of large amounts of sea spray^16^, which was implemented this into the concept of rapid intensification and the aerodynamic drag well^11^.

A complete investigation of the effect of bio-surfactants on the tropical cyclone intensity and intensification is not feasible at this point due to the complexity of the problem and scarcity of the field data and is beyond the scope of this paper. The extent of the surfactant effect on tropical cyclones might be qualitatively estimated by correlating the tropical cyclone intensity changes and the presence of algal blooms identified from color satellite imagery, assuming that algal blooms are favorable for the generation of bio-surfactants. The presence of bio-surfactants, as well as anthropogenic surfactants (oil spills and dispersants), can also be identified from dual-polarimetric synthetic aperture radar (SAR) imagery using the co-polarized phase difference filter^69–71^, which may contribute to better understanding the potential effect of surfactants on tropical cyclone intensity and intensification.

## Conclusions

This work, through the use of laboratory and computational fluid dynamics (CFD) model experiments, has demonstrated, for the first time, the increase of the sea spray generation and change in size distribution under tropical cyclone conditions in the presence of surfactants. As surfactants increase spray generation and change the size distribution, they influence heat and momentum fluxes to the atmosphere, potentially affecting tropical cyclone intensity.

The global distribution of bio-surfactants on the earth is virtually unknown at this point^59^. We imply that it is related to biological primary productivity and chlorophyll concentration^72,73^. Future considerations should include using satellite imagery to associate primary productivity along tropical cyclone paths, in an effort to further develop the potential relationship between biological surfactants and tropical cyclone intensity. Including evaporation and heat fluxes in our CFD modeling would greatly increase the understanding of these relationships, which are difficult to observe and measure in the field and laboratory. In the ocean under tropical cyclone conditions, surfactants on the sea surface affect heat, energy, and momentum exchange through altered size distributions of sea spray, with possible consequences for tropical cyclone intensification or decline, particularly in biologically productive areas and the areas affected by oil spills and dispersants. The approach developed in this work potentially provides an opportunity to improve the sea spray generation function, without and with surfactants. This problem, however, is beyond the scope of this paper.

## Methods

### Laboratory experiment

In this section the experimental design relevant to this work will be briefly described. A detailed description of the experiment can be found in Soloviev et al.^74^. A laboratory experiment was conducted at the University of Miami Rosenstiel School of Marine and Atmospheric Science (RSMAS), using the Air-Sea Interaction Saltwater Tank (ASIST) at the SUSTAIN facility. The tank is 15 m long by 1 m width by 1 m in height, and has an acrylic glass exterior, which is transparent and allows for equipment to be placed outside the tank rather than within. The water level was set to 0.42 m for this experiment.

The ASIST tank contains various equipment to simulate ocean conditions. The wave generator produces waves with frequencies between 0.25 and 3 Hz, and amplitudes between 0 and 0.1 m. The wind generator is able to produce winds up to 40 m s^−1^ (recalculated to a 10 m height), while the current generator can produce current speeds up to 0.5 m s^−1^. For our experiment, we set the wind speed (extrapolated to 10 m height) to 40 m s^−1^. Temperature is also controlled and can be kept anywhere between 5 and 40° C. For our experiments, the water temperature was 23.2° C and 24.3° C. The air in the tank is circulated with either an open or closed loop. Fresh air from the atmosphere is captured and expelled after passing through the tank. In the closed loop option, which was used for this experiment, the air is maintained in the tank. Air temperatures were set to 24.5° C and 24.6° C for our experiments. The experiments were done using 10 *μ*m filtered seawater. For all of the experiments, the salinity of the seawater was ~ 33 psu.

A Digital Laser Elevation Gauge (DLEG), consisting of a line-scan camera and laser beam that crossed the tank at the water surface, was set up on the outside of the tank. Additionally, two Argon-ION air-cooled lasers were used, which contained beam splitters and mirrors. This allowed for six vertical beams to be focused on any point in the tank, which is crucial as these beams and line-scan cameras detect the water surface. In order to make the beams easier to see, Fluorescein was added to the water in the tank. The line-scan cameras had a 1024-pixel resolution, and a 250 Hz sampling rate was used for the experiment. Using this equipment allowed for an accurate surface elevation measurement, to 0.2 mm resolution. A *Hisense* camera was also synchronized with the lasers to record images. To capture spray droplets, a collimated light beam was used. This beam was focused through a diffusing screen to reduce its intensity. The *Dantec* camera was then placed opposite from this diffused beam. Images of spray droplets were focused on a single plane, located at the center of the ASIST tank. During the experiment, pairs of images were taken 500 μm apart^75,76^.

Surfactant trials were conducted after clean water experiments, and the water in the tank was switched between each trial. In order to introduce surfactants, either Oleic acid or oleyl alcohol was used, which are both insoluble in water. Both were diluted in 95% ethanol, oleic acid with an 8 mmol/liter ethanol concentration, and oleyl alcohol with a 3 mmol/liter ethanol concentration. During each trial, 60 ml of the solution was added to the tank using a syringe from the upwind side of the tank. The surfactant plume passed by the measurement area with the surface current created by the applied wind stress.

### Computational fluid dynamics model

ANSYS FLUENT 19.2 was used to model the effect of surfactants on the generation of sea spray under tropical cyclone conditions. For further consideration of the theory behind FLUENT models, please see the *ANSYS Fluent Theory Guide 19.2*^77^.

The model was run in parallel on 264 processors on a 564 core HPC Linux cluster with 12 compute nodes. We used a pressure-based, transient, 1^st^ order implicit solver and a Large Eddy Simulation to model turbulence. Operating conditions were set to 293.15 K, 9.81 m s^−1^ gravity, and 101,325 Pa atmospheric pressure. We specified the materials in our domain as water with a bulk temperature T = 298 K, density ρ = 1000 kg m^−3^, viscosity *μ* = 0.001003 kg·m^−1^·s^−1^, and specific heat *cp* = 4182 J kg^−1^ K^−1^, and air with bulk temperature T = 298.15 K, density ρ = 1.225 kg m^−3^, viscosity *μ* = 1.7894e^−05^ kg·m^−1^·s^−1^ , and specific heat *cp* = 4182 J kg^−1^ K^−1^.

The Volume of Fluid to Discrete Phase Model (VOF-to-DPM) is an innovative model from Fluent that allowed us to resolve sea spray down to 100 μm of radius through dynamic mesh adaption. The model combines two of Fluent’s models: Volume of Fluid and Discrete Phase Model. The DPM tracks particles which are formed through specified parameters by Lagrangian tracking methods. We set interaction with the continuous phase, unsteady particle tracking, secondary breakup, and track with fluid flow time-step within this model. We injected particles at a very high start and stop time as a placeholder for particles created using the VOF-to-DPM transition. The particle material was set to pure liquid water. The VOF model allows for multiphase modeling, we set two phases (air and water) with explicit formulation and sharp/dispersed interface modeling. Waves with a 0.005 m height and 0.05 m length were set up using the open channel flow feature of this model. The phase interactions were set to a constant surface tension, depending on whether surfactants were introduced, or the water was clean. A sensitivity model experiment with a 50% reduction of surface tension resulted in a stronger effect of surfactants on spray generation than a 25% reduction. The 25% reduction was selected based on the King et al.^59^ report on surface tension measurements in the coastal North Sea in the presence of surfactants. The surface tension to introduce surfactants was therefore set to 0.054 N/m, while the clean water’s surface tension was set to 0.072 N/m.

The phase model transitions were set to transfer water parcels tracked by VOF to Lagrangian particles set by DPM. This transition was specified to allow parcels that were within the volume-equivalent sphere diameter range of 0–0.005 m and upper limits of asphericity as calculated by the radius standard deviation of 0.5 and radius surface orthogonality of 0.5. Lumps that exceeded cell volume by a factor of 10 were split into parcels. The mesh was coarsened immediately after lump conversion to save computational power. Mesh adaption is key to the transition process. Mesh adaption was set based on the curvature of the volume fraction of water within each mesh cell. It was applied to coarsen and refine every two time-steps, up to 10 levels per cell. According to Eggers^78^, the timescale for breakup of free surface flows is on the order of 10^−2^ s. The time step in the ANSYS Fluent model was in the range of 10^−9^–10^−6^ s, which is several orders of magnitude less than the timescale for breakup of free surface flows. This is more than enough to capture any fast transients during the interface breakup.

The model domain was created in ANSYS Workbench and consisted of a 0.1 m (x) by 0.1 m (y) by 0.05 m (z) box with an initial mesh size of 0.002 m. This mesh was then adapted, as previously described, allowing for remeshing down to tens of micrometers in order to confidently resolve spray particles starting from a 100 μm radius. Boundary conditions were set to zero shear on lateral sides and bottom of the domain. The domain contained air and water, with the water being initialized with a wavy interface from the inlet. Wind stress was applied at the top of the domain. This was set according to the strength of tropical cyclone conditions being modeled, 4 Nm^−2^ for Category 1, 10 Nm^−2^ for Category 3, and 20 Nm^−2^ for Category 5. The model was run to allow the initialized waves to set up before setting periodic boundary conditions at the inlet and outlet, which permitted the waves to propagate through the domain.

### Data analysis

Images from the University of Miami laboratory experiment were first processed using the *Dantec Dynamics* shadow imaging software package to remove some background noise and focus on the spray droplets taken during the experiment. The images were then analyzed using MATLAB to determine how many pixels each spray droplet image was in the x and y direction. The average diameter was calculated for each spray droplet (1 pixel = 42 μm) to determine the size distribution (in terms of radius). The diameters of the spray particles produced by the CFD models were exported from ANSYS Fluent as a data file. Data files containing CFD model spray diameters and laboratory experiment spray diameters were analyzed in MATLAB using histograms, plots, and normalization to calculate spray radius probability distributions. For model verification, probability distribution plots were created in MATLAB from the laboratory data.

## Data Availability

The datasets generated during and/or analyzed during the current study are available from the corresponding author on reasonable request.
