# Author Correction: Potential effect of bio-surfactants on sea spray generation in tropical cyclone conditions

**DOI:** 10.1038/s41598-021-88354-w

**Published:** 2021-04-15

**Authors:** Breanna Vanderplow, Alexander V. Soloviev, Cayla W. Dean, Brian K. Haus, Roger Lukas, Muhammad Sami, Isaac Ginis

**Affiliations:** 1 Halmos College of Arts and Sciences, Nova Southeastern University, Dania Beach, FL USA; 2 University of Miami, Miami, FL USA; 3 University of Hawaii, Honolulu, HI USA; 4 Ansys, Inc., Houston, TX USA; 5 University of Rhode Island, South Kingstown, RI USA

Correction to: *Scientific Reports* 10.1038/s41598-020-76226-8, published online 4 November 2020

The Data Availability statement in this Article is incorrect.

“The datasets generated during and/or analyzed during the current study are available from the corresponding author on reasonable request.”

should read:

“Data are publicly available through the Gulf of Mexico Research Initiative Information & Data Cooperative (GRIIDC) at https://data.gulfresearchinitiative.org (DOI:10.7266/2QM6R71Y).”

